# Global prevalence of violence against women with disabilities: protocol for a systematic review and meta-analysis

**DOI:** 10.1136/bmjopen-2025-102746

**Published:** 2025-11-04

**Authors:** Laura Campo-Tena, LynnMarie Sardinha, Sarah R Meyer, Claudia García-Moreno

**Affiliations:** 1 Institute of Criminology, University of Cambridge, Cambridge, UK; 2 Department of Sexual and Reproductive Health and Research, World Health Organization, Geneva, Switzerland

**Keywords:** Prevalence, Systematic Review, Gender-Based Violence, Disabled Persons, Risk Factors

## Abstract

**Abstract:**

**Introduction:**

Violence against women (VAW) is a public health, gender equality and human rights issue, with women with disabilities facing heightened risks due to intersecting discrimination. However, research on violence against people with disabilities often lacks sex-disaggregated data and primarily focuses on intimate partner violence, neglecting other perpetrators like family members or caregivers and leading to potential underestimation of its prevalence.

**Methods and analysis:**

This protocol outlines the methods for a systematic review and meta-analysis to estimate the global prevalence of VAW with disabilities, focusing on intimate partner violence by intimate partners, domestic violence and sexual violence by any perpetrator. The review will adhere to the Preferred Reporting Items for Systematic Reviews and Meta-Analyses guidelines and will include population-based quantitative studies focusing on women with disabilities aged 15 and older. 12 databases will be searched, and records will be screened by two independent reviewers. The risk of bias will be assessed. The global prevalence and pooled ORs comparing women with and without disabilities will be calculated.

Findings will contribute to global efforts in addressing VAW with disabilities, informing the development of targeted, evidence-based policies and programmes and ensuring that interventions are responsive to their specific needs and circumstances.

**Ethics and dissemination:**

Ethics approval is not required, as this review analyses previously published data. Findings will inform ongoing World Health Organization (WHO)/Human Reproductive Programme (HRP) work on strengthening the measurement of VAW with disabilities and will be disseminated through a peer-reviewed publication, conference presentations and sharing with relevant organisations.

**PROSPERO registration number:**

CRD42023427512.

**STRENGTHS AND LIMITATIONS OF THIS STUDY**

- This review will use a comprehensive and structured search strategy across multiple databases.
- Nationally and sub-nationally representative studies will be included.
- A standardised process will be applied for data extraction and risk of bias assessment.
- Findings will be synthesised using systematic approaches to analysis.
- The review may be limited by heterogeneity in measurement tools and definitions across studies.

## Background

 Violence against women (VAW) is a public health concern, a gender inequality issue and a violation of women’s fundamental human rights. It encompasses any form of gender-based violence that causes or has the potential to cause physical, sexual or psychological harm or suffering to women, including coercion, threats or deprivation of liberty, in private or public settings.^1^ Globally, about one in three women have been subjected to either physical and/or sexual intimate partner violence or non-partner sexual violence in their lifetime, and research shows that the impact of violence can be far-reaching, affecting physical, mental, sexual and reproductive health.^1 2^ When gender intersects with other factors of discrimination, such as disability, women are at even higher risk of experiencing violence.^1 3 4^

Disability is a multifaceted, diverse and evolving concept that encompasses physical, mental, intellectual, sensory and socioemotional long-term impairments and that can range in severity.^5^,^7^ The state of disability arises from a combination of health conditions and environmental factors.^8^ Globally, around 1.3 billion people (about 16% of the global population) experience significant disability, such as blindness or spinal cord injury.^9^ Prevalence of disabilities is higher among women (18.4%) compared with men (14.3%).^9^ In low- and middle-income countries, the gender imbalance in disability is even more pronounced, with women accounting for three-quarters of all individuals with disabilities.^10^

In recent years, the question of whether women with disabilities are at higher risk and are disproportionately affected by violence has received increased attention.^11^,^15^ Research suggests that interpersonal violence prevalence is higher in individuals with disabilities, compared with people without disabilities^7 16^ and that different types of disabilities may result in different risk and prevalence estimates. Mental illness, for instance, has been linked to increased vulnerability to violence.^16^ Following Meyer and colleagues,^17^ common mental disorders will be excluded from this review due to the extensive and well-established body of evidence linking VAW with these conditions, which has been supported by numerous systematic reviews and meta-analyses.^18^,^20^

Most existing studies on violence against people with disabilities do not disaggregate data by sex,^16 21^ limiting our knowledge of the prevalence and nature of VAW with disabilities.^22 23^ While all people with disabilities may be at increased risk of violence, existing evidence suggests that women with disabilities are at greater risk of and experience higher levels of violence, including violence by intimate partners or other family members and sexual violence by any perpetrator globally compared with men.^4 24^ Discrimination, stigma and misconceptions about disability, as well as restrictive gender and social norms, can increase the exposure of women with disabilities to violence and influence their experience of it.^25^ Difficulties in escaping violence, associated with having a poor or very poor health status, may increase the vulnerability of women with disabilities.^26^ Women with disabilities are also subjected to and vulnerable to specific forms of violence. Current estimates are likely substantial underestimates as women with disabilities are often under-represented in research, including population-based surveys, and may experience disability-specific types of violence that are rarely captured.^23^ This increased risk for violence in women is compounded by the fact that those with disabilities face more barriers to accessing healthcare and other services than women without disabilities.^27^

Violence perpetrated by intimate partners is one of the most common forms of VAW globally^1^ and remains a prevalent form of violence for women with disabilities.^3^ Additionally, perpetrators may include non-partners—such as those in positions of trust—who may account for a substantial share of violence against this population.^28^ Yet, caregivers and other relevant perpetrators who have an increased likelihood of perpetrating abuse against persons with disabilities are frequently omitted in current research and survey measures.^29^ For example, a scoping review on the measurement of VAW and disability found that more than one-third of the included studies focused solely on intimate partner violence.^17^

Target 5.2 of the 2030 Sustainable Development Goals to eliminate all forms of violence against all women and girls was adopted by the United Nations General Assembly in 2015 under Goal 5 on gender equality.^30^ This commits governments to ensure that efforts to prevent, address and eliminate VAW are inclusive and reach the most marginalised groups, including women with disabilities, who often face multiple layers of discrimination and exclusion.

Despite the growing interest and recognition of the importance of the issue, there is still a limited understanding of disability as a risk factor for women’s experience of violence.^17^ With the expected rise in disability rates in the coming years due to various demographic and epidemiological changes^9^ and the well-evidenced negative impacts of violence on women’s physical and mental health and well-being, there is an urgent need for more and better-quality data on the prevalence and the risk of VAW with disabilities. Understanding the magnitude, extent and nature of violence that women with disabilities are subjected to would allow more tailored prevention strategies and response/services and programmes designed to better address the specific needs of women with disabilities. Robust data on this issue are also a powerful advocacy tool for greater investment in preventing and addressing such violence.

Several gaps in research have motivated the current study, including the lack of sex-disaggregated data on violence against people with disabilities—and, therefore, limited evidence specific to women—insufficient information on the types, severity and duration of the violence they experience and its perpetrators, and a lack of data on how different types of disabilities may be associated with distinct forms of violence.^23^

Previous systematic reviews have explored violence against people with disabilities. For instance, Hughes and colleagues^16^ examined violence against adults with disabilities, though without disaggregating findings by sex. Other reviews have focused on intimate partner violence among women with disabilities,^3^ on interventions for the primary prevention of VAW with disabilities^31^ and on the measurement in a scoping review of VAW and disability.^17^ However, no previous systematic reviews have focused on exploring VAW with disabilities specifically.

By addressing these gaps, the present review will contribute to the literature by offering the first comprehensive and quantitative synthesis of global prevalence and risk of VAW with disabilities. Unlike previous reviews, it will integrate multiple forms of violence (intimate partner, domestic violence and sexual violence by any perpetrator, covering various potential perpetrators and including family members and caregivers) and systematically incorporate sex-disaggregated data. In doing so, this study will not only provide more complete prevalence estimates but also generate evidence that can guide policy, prevention and training efforts.

### Aim and objectives

The aim of the systematic review and meta-analysis will be to assess the prevalence and risk of VAW with disabilities at a global level in population-based studies. To ensure both comprehensiveness and comparability with existing evidence, our scope includes physical, psychological, sexual and other forms of violence, while focusing on three main perpetrator categories: intimate partners, domestic violence and sexual violence by any perpetrator, including family members and caregivers. This approach builds on but also extends previous reviews, which often concentrated exclusively on intimate partner violence or did not disaggregate by sex.

The review will have the following objectives: (1) to identify primary observational studies on the prevalence of VAW with disabilities; (2) to evaluate the quality of the studies; (3) to synthesise the evidence on the prevalence of VAW with disabilities; (4) to calculate pooled ORs with 95% CIs for the risk of violence in women with disabilities compared with women with no disabilities and (5) to identify any knowledge gaps and research priorities related to this topic.

### Review questions

This study will seek to address the following research questions: (Q1) What are the global prevalence estimates for VAW with disabilities?; (Q2) Do prevalence estimates vary by the type of disability, type of violence or world region? and (Q3) Is violence more prevalent among women with disabilities compared with women with no disabilities?

## Methods

This review follows and builds on the methodology used in a previous systematic review and meta-analysis conducted by Hughes and colleagues^16^ on the prevalence and risk of violence against adults with disabilities. However, our planned review will focus on the prevalence and risk of VAW with disabilities, specifically.

The review will follow the Preferred Reporting Items for Systematic Reviews and Meta-Analyses (PRISMA) guidelines. This protocol adheres to The PRISMA statement for Protocols (PRISMA-P) and has been registered with PROSPERO International Prospective Register of systematic reviews. Although preliminary work has been carried out, the review is scheduled to officially begin in September 2025 and conclude by May 2026.

### Search strategy

#### Databases

We will search in the following bibliographic databases: Medline, PsycINFO, CINAHL, International Bibliography of the Social Sciences, ASSIA, ERIC, Sociological Abstracts, Cochrane Library, Embase, National Criminal Justice Reference System Abstracts Database, Social Sciences Citation Index, WHO Global Health Library (LILACS, IBCS, BDENF, African Index Medicus, Index Medicus for Eastern Mediterranean Region (IMEMR), Latin American and Caribbean Center on Health Sciences Information (PAHO) Library and Western Pacific (WPRO).

#### Languages

The systematic search for relevant studies will be conducted in English. No language restrictions will be applied to the search results obtained. In cases where citations are obtained in languages other than English, relevant translations will be conducted by a member of the team who can speak the language in question fluently, or alternatively, will be translated using Google Translate for screening and data extraction.

#### Search terms

The selection of search terms for the systematic search was informed by the search strategies from two published reviews. First, Hughes *et al*’s systematic review^16^ was consulted. Second, we referred to the scoping review conducted by Meyer and colleagues^17^ and the systematic review conducted by García-Cuéllar and colleagues.^3^ Table 1 provides details on the proposed PubMed search terms. These terms will serve as a reference and will be adapted as needed to account for the specific requirements of each database (see online supplemental file 1).

**Table 1:** PubMed search strategy

	Terms
1	“Intellectual disability”[MeSH] OR “Communication disorders”[MeSH] OR “Developmental disabilities”[MeSH] OR “Mentally Disabled persons”[MeSH] OR “Disabled persons”[MeSH] OR “Disab*”[TIAB] OR “Intellectual* Disab*”[TIAB] OR “Physical* disab*”[TIAB] OR “Intellectual* Handicap*”[TIAB] OR “Intellectual* impair*”[TIAB] OR “Mental* disab*”[TIAB] OR “Physical* disab*”[TIAB] OR “Physical* handicap*”[TIAB] OR “Physical* impair*”[TIAB] OR “Vision impair*”[TIAB] OR “Visual impair*”[TIAB] OR “Hearing impair*”[TIAB] OR “Deaf*”[TIAB] OR “Blind*”[TIAB] OR “Motor Disab*”[TIAB] OR “Neuromotor Disab*”[TIAB] OR “Mobility impair*”[TIAB] OR “Functional* impair*”[TIAB]
2	“Domestic violence”[MeSH] OR “Intimate Partner Violence”[MeSH] OR “Battered women”[MeSH] OR “Violence”[MeSH] OR “Aggression”[MeSH] OR “Spouse abuse”[MeSH] OR “Physical Abuse”[MeSH] OR “Rape”[MeSH] OR “Assault”[TIAB] OR “Sexual abuse”[TIAB] OR “sexual assault”[TIAB] OR “Rape”[TIAB] OR “Psychological abuse”[TIAB] OR “Psychological violence”[TIAB] OR “Emotional abuse”[TIAB] OR “Emotional violence”[TIAB] OR “Neglect”[TIAB] OR “Economic abuse”[TIAB] OR “Financial abuse”[TIAB] OR “Verbal abuse”[TIAB] OR “Violence against women”[TIAB] OR “Abused women”[TIAB] OR “Intimate terrorism”[TIAB] OR “Marital rape”[TIAB] OR “Wife beating”[TIAB] OR “Relationship aggression”[TIAB] OR “Restraint”[TIAB] OR “Reproductive coercion”[TIAB] OR “Pregnancy coercion”[TIAB] OR “Contraceptive coercion”[TIAB] OR “Forced contraception”[TIAB] OR “Forced sterilization”[TIAB] OR “Forced sterilisation”[TIAB]
3	“Prevalence”[Mesh] OR “Incidence”[Mesh] OR “Prevalence”[TIAB] OR “Incidence”[TIAB] OR “Risk*“[TIAB] OR “Experienc*“[TIAB] OR “Expos*"[TIAB]
4	“Women”[MeSH] OR “Female”[MeSH] OR “Wife”[TIAB] OR “Spouses”[MeSH] OR “Wives”[TIAB] OR “Partner*” [TIAB] OR “Spouse*”[TIAB]
1 AND 2 AND 3 AND 4	

### Selection process and criteria

Bibliographic database citations that result from the predefined search strategy will be imported to Covidence.^32^ Duplicates will be removed from the list of retrieved citations before the screening process.

Screening will be conducted following the pre-established inclusion and exclusion criteria (table 2) using Covidence.^32^ We will divide the screening process into two stages: (1) by title and abstract and (2) by full-text publications. In cases where no online copy of citations included for full-text screening is available, we will contact the authors, sending a maximum of two reminders, to request the publication’s full text.

**Table 2:** Inclusion and exclusion criteria for study selection

	Inclusion	Exclusion
Participants	Adult women (aged 18 years and older) and adolescent girls (15–17) with disabilities. Studies including men in the sample will be considered if disaggregation by sex is provided.	Women without disabilities or younger than 15 years. Samples only formed by men, or formed by men and women without providing disaggregation by sex.
Outcomes	IPV, domestic violence and/or sexual violence by any perpetrator.	Other forms of violence measured (eg, street violence) or no form of violence measured.
Types of disability	Physical, intellectual and sensory disabilities.	Common mental disorders.
Study design and type of publication	Cross-sectional, case control or longitudinal studies. Peer-reviewed and grey literature (eg, PhD thesis, study reports).	Pilot studies, narrative and systematic reviews, meta-analyses, conference proceedings, case reports, qualitative studies, editorials, opinion papers and letters.
Study focus/region	Nationally or sub-nationally representative population-based studies (Q1, Q2, Q3). Non-representative studies of specific subgroups of women with disabilities (eg, women with cognitive disabilities). These will not contribute to pooled global prevalence (Q1) but will be included for subgroup analyses (Q2). Comparative studies of women with and without disabilities that report ORs or other comparative measures (Q3).	Studies with no clear denominator. Studies on samples from clinic, care home or other institutional settings.
Data reported	Report either prevalence or ORs, or raw data to enable their calculation.	Do not provide quantitative data on prevalence estimates or risk of violence.
Reported timeframe	Past 12 months. Lifetime. Perinatal period.	Other than past 12 months, lifetime or perinatal period (eg, last 2 years).
Response rate	50% and over.	Less than 50%.
Timeframe of searches	From 1 Jan 1990.	Before 1 Jan 1990.
Language	No language restrictions.	–
Same-source surveys	When same-source surveys are identified, only data considering different outcomes will be included. If two or more studies are based on the same data source, we will prioritise the one with the highest research quality.	

IPV, intimate partner violence.

Two independent reviewers (LC-T and SRM) will assess the retrieved citations according to the pre-defined selection criteria (table 2). Any disagreements will be discussed, involving a third researcher (LMS or CG-M), if necessary, to reach agreement. Cohen’s kappa will be used to test for reporting levels of inter-rater reliability, using Covidence.^32^ A flowchart will be used to illustrate the study selection process. Reasons for excluding citations at the full-text stage will be reported as per the PRISMA-P.

### Data extraction and management

The data extraction will be conducted by one data analyst (LC-T) into a template matrix and will undergo rigorous independent quality checks by a second data analyst in the team (SRM). Any disagreements will be discussed and, if necessary, will involve a third member of the team (LMS or CG-M).

The data categories for extraction are informed by Hughes and colleagues^16^ and have been re-assessed in consultation with the WHO to ensure comprehensive coverage of relevant variables for the purposes of our review and to support potential future analyses. The main data categories will include:

- Study identifier: study title, author, publication year.
- Geographical information: Global Burden of Disease super region, Global Burden of Disease region, country of publication, iso 3 codes.
- Main characteristics of studies included: start and end date of data collection, study design, quality assessment score, whether study took place in an urban and/or rural area, whether fieldworkers/interviewers were trained, study setting, sample size.
- Participant characteristics: mean age, age range, whether breakdown by age is provided when reporting results, and if so, what is the breakdown provided, sex of participants (if female 100%, or what is the percentage of males included).
- Comparison group, if any (eg, women with disabilities vs women without disabilities).
- Data on prevalence of VAW with disabilities.
- Type of disability reported (non-specific, physical, sensory, intellectual) and, if available, timeframe of disability.
- Type of violence reported (physical, psychological, sexual, any violence, other) and timeframe used (past 12 months, lifetime or perinatal).
- Type of perpetrator (eg, intimate or dating partner, spouse, ex-intimate or ex-dating partner, ex-spouse, relative, formal caregiver, any).
- Instrument name and number of items used to measure violence and disability.
- Response rate.
- Adjusted and/or unadjusted ORs, 95% CIs, p values.

### Risk of bias assessment

The *risk of bias tool*,^33^ developed to assess the quality of population-based prevalence studies through 10 items, will be used to assess the risk of bias of the included studies that examine prevalence rates. For studies that also examine the risk of violence for women with disabilities, we will consider four additional items adapted for this purpose and previously used in Hughes and colleagues.^16^ Two reviewers will independently assess all included studies and resolve any discrepancies through consensus.

### Proposed statistical analysis

We will conduct a meta-analysis, if appropriate, using a random-effects model to account for between-study variability.

For studies reporting prevalence rates, we will extract raw proportions and compute pooled prevalence estimates with 95% CIs. Lifetime and past 12 month prevalence estimates will be analysed separately.

For studies that include two comparison groups (ie, women with disabilities and women without disabilities), we will calculate extracted ORs and their 95% CIs. We will calculate pooled ORs, also using a random-effects model, to assess the relative risk of violence among women with disabilities compared with women without disabilities.

To assess the heterogeneity of the studies included, we will report I² statistics, which quantifies the percentage of variation across studies due to heterogeneity rather than chance. We will also compute the Cochran’s Q test and τ² (tau-squared) statistic to further assess between-study variance. If substantial heterogeneity is detected (I²>50% or p<0.10 in Cochran’s Q test), we will explore potential sources of heterogeneity through subgroup analyses and, if feasible, meta-regression. Subgroup analyses will examine factors such as geographical region, type of violence and type of disability.

To assess publication bias, we will use funnel plots, Egger’s regression test and Begg’s rank correlation test, provided that at least 10 studies are included in the analysis. If asymmetry is detected, we will consider using the trim-and-fill method to adjust for potential small-study effects. All analysis will be conducted using Comprehensive Meta-Analysis (V.4).^34^ Forest plots will be generated to depict either prevalence proportions or ORs with corresponding CIs for each study and the combined effect size, to provide an overview of the results. Where appropriate, univariate analyses will be used to test the individual association of several covariates with pooled estimates, mainly geographical region and violence outcome.

If a meta-analysis is inappropriate for this study, data will be synthesised and presented using a narrative approach and supplemented by simple descriptive statistics to summarise key trends.

## Ethics and dissemination

No ethics approval is required for this systematic review, as it will involve the analysis of previously published data and will not involve human participants.

Findings will feed into ongoing work by WHO/Human Reproductive Programme (HRP) on strengthening the measurement of VAW with disabilities. The results of the study will be published in a peer-reviewed journal. They will also be presented at relevant conferences and shared with key organisations addressing VAW with disabilities.

## Discussion

VAW is a pressing public health, gender equality and human rights concern, with women with disabilities experiencing heightened vulnerability due to intersecting forms of discrimination.^1 3 4^ Yet, evidence in this area has been limited, with much of the existing research lacking sex-disaggregated data and disproportionately focusing on intimate partner violence, often overlooking other perpetrators such as family members or caregivers.^3 11 16 21^ This review will address these gaps by systematically estimating the global prevalence of violence among women with disabilities, encompassing domestic violence and sexual violence by anyone, in addition to intimate partner violence. By synthesising evidence from population-based quantitative studies and generating pooled prevalence estimates and ORs, this review will provide a comprehensive understanding of the scope of VAW with disabilities and lay the groundwork for strengthening future research, policy and practice in this area. This protocol outlines the rationale, objectives and methods for conducting such a systematic review and meta-analysis. To the best of our knowledge, this will be the first study of its kind to be published, providing an analysis of this issue on a global scale.

A strength of this review is its inclusion of perpetrators beyond intimate partners, expanding on the approach taken in previous similar reviews.^3 11^ While women with disabilities are nearly twice as likely to report violence from an intimate partner, compared with women without disabilities,^11^ they are also vulnerable to sexual and other forms of violence from a variety of individuals such as other family members or other caregivers.^35 36^ The omission of perpetrators, other than intimate partners, in the measurement of VAW with disabilities may result in an under-estimation of the prevalence.^23 37^

Furthermore, this review expands on previous reviews on violence against people with disabilities that did not disaggregate findings by sex.^16 21^ Combining data for men and women hinders our understanding of VAW with disabilities and the extent to which disability increases the risk of violence. By focusing on women with disabilities, this study seeks to elucidate these interactions and contribute to a more nuanced understanding of the issue.

Although recommendations will be presented once the findings of this review are available, some future research priorities can already be anticipated as they motivated the current study. First, studies on violence against people with disabilities should systematically include sex-disaggregated data to better understand differential risks and vulnerabilities. Second, research should expand beyond intimate partner violence to capture other forms of abuse, including those perpetrated by family members, caregivers and institutional actors, in order to more comprehensively estimate prevalence and patterns. Finally, findings from this review may inform the design of training programmes for researchers and practitioners to strengthen methodological rigour and promote more inclusive and context-sensitive approaches to studying violence against people with disabilities.

Findings from this systematic review and meta-analysis are expected to enhance our understanding of the increased risks faced by women with disabilities, thereby informing the development of targeted, evidence-based policies and programmes. Ultimately, the review aims to contribute to global efforts in preventing violence and improving support services for women with disabilities, ensuring that interventions are responsive to their specific needs and circumstances.

## Supplementary material

10.1136/bmjopen-2025-102746 online supplemental file 1
