# Supplementary material for: Global prevalence of violence against women with disabilities: protocol for a systematic review and meta-analysis
Source: BMJ Open. 2025 Nov 4;15(11):e102746. doi: 10.1136/bmjopen-2025-102746 (PMC12588038; doi:10.1136/bmjopen-2025-102746)
Supplement: online supplemental file 1 [file bmjopen-15-11-s001.docx]

***Global Prevalence of Violence Against Women with Disabilities: Protocol for a Systematic Review and Meta-Analysis***

**Supplementary File - Search Strategy**

| ***Search number*** | ***Terms*** |
| --- | --- |
| **Pubmed** | |
| 1 | "Intellectual Disability"[MeSH] OR "Communication Disorders"[MeSH] OR "Developmental Disabilities"[MeSH] OR "Mentally Disabled Persons"[MeSH] OR "Disabled Persons"[MeSH] OR “Disab*”[TIAB] OR "Intellectual* Disab*"[TIAB] OR "Physical* disab*"[TIAB] OR "Intellectual* Handicap*"[TIAB] OR "Intellectual* impair*"[TIAB] OR "Mental* disab*"[TIAB] OR "Physical* handicap*"[TIAB] OR "Physical* impair*"[TIAB] OR "Vision impair*"[TIAB] OR "Visual impair*"[TIAB] OR "Hearing impair*"[TIAB] OR "Deaf*"[TIAB] OR "Blind*"[TIAB] OR "Motor disab*"[TIAB] OR "Neuromotor disab*"[TIAB] OR "Mobility impair*"[TIAB] OR "Functional* impair*"[TIAB] |
| 2 | "Domestic Violence"[MeSH] OR "Intimate Partner Violence"[MeSH] OR "Battered Women"[MeSH] OR "Violence"[MeSH] OR "Aggression"[MeSH] OR "Spouse Abuse"[MeSH] OR "Physical Abuse"[MeSH] OR "Rape"[MeSH] OR "Assault"[TIAB] OR "Sexual abuse"[TIAB] OR "Sexual assault"[TIAB] OR "Rape"[TIAB] OR "Psychological abuse"[TIAB] OR "Psychological violence"[TIAB] OR "Emotional abuse"[TIAB] OR "Emotional violence"[TIAB] OR "Neglect"[TIAB] OR "Economic abuse"[TIAB] OR "Financial abuse"[TIAB] OR "Verbal abuse"[TIAB] OR "Violence against women"[TIAB] OR "Abused women"[TIAB] OR "Intimate terrorism"[TIAB] OR "Marital rape"[TIAB] OR "Wife beating"[TIAB] OR "Relationship aggression"[TIAB] OR "Restraint"[TIAB] OR "Reproductive coercion"[TIAB] OR "Pregnancy coercion"[TIAB] OR "Contraceptive coercion"[TIAB] OR "Forced contraception"[TIAB] OR "Forced sterilization"[TIAB] OR "Forced sterilisation"[TIAB] |
| 3 | "Prevalence"[MeSH] OR "Incidence"[MeSH] OR "Prevalence"[TIAB] OR "Incidence"[TIAB] OR "Risk*"[TIAB] OR "Experienc*"[TIAB] OR "Expos*"[TIAB] |
| 4 | "Women"[MeSH] OR "Female"[MeSH] OR "Wife"[TIAB] OR "Spouses"[MeSH] OR "Wives"[TIAB] OR "Partner*"[TIAB] OR "Spouse*"[TIAB] |
| 1 AND 2 AND 3 AND 4 |  |
| **PsycINFO** | |
| 1 | DE "Disab*" OR DE "Intellectual Development Disorder" OR DE "Communication Disorders" OR  DE "Developmental Disabilities" OR DE "Physically Disabled Persons" OR DE "Mentally Disabled Persons" OR  (disab* OR "intellectual disability" OR "communication disorders" OR "developmental disabilities" OR  "disabled persons" OR "intellectual* disab*" OR "physical* disab*" OR "intellectual* handicap*" OR  "intellectual* impair*" OR "mental* disab*" OR "physical* handicap*" OR  "physical* impair*" OR "vision impair*" OR "visual impair*" OR "hearing impair*" OR "deaf*" OR "blind*" OR  "motor disab*" OR "neuromotor disab*" OR "mobility impair*" OR "functional* impair*").ti,ab,kw |
| 2 | DE "Domestic Violence" OR DE "Spouse Abuse" OR DE "Partner Abuse" OR DE "Sexual Assault" OR  DE "Emotional Abuse" OR DE "Psychological Abuse" OR DE "Economic Abuse" OR  ( "domestic violence" OR "intimate partner violence" OR "battered women" OR violence OR aggression OR  "spouse abuse" OR "physical abuse" OR "rape" OR "assault" OR "sexual abuse" OR "sexual assault" OR  "psychological abuse" OR "psychological violence" OR "emotional abuse" OR "emotional violence" OR  neglect OR "economic abuse" OR "financial abuse" OR "verbal abuse" OR "violence against women" OR  "abused women" OR "intimate terrorism" OR "marital rape" OR "wife beating" OR "relationship aggression" OR  restraint OR "reproductive coercion" OR "pregnancy coercion" OR "contraceptive coercion" OR  "forced contraception" OR "forced sterilization" OR "forced sterilisation").ti,ab,kw |
| 3 | DE "Prevalence" OR DE "Incidence" OR DE "Risk Factors" OR  (prevalence OR incidence OR risk* OR experienc* OR expos*).ti,ab,kw |
| 4 | DE "Women" OR DE "Human Females" OR DE "Wives" OR  (women OR woman OR female* OR girl* OR wife OR wives OR spouse* OR partner* OR "female partner").ti,ab,kw |
| 1 AND 2 AND 3 AND 4 |  |
| **CINAHL** | |
| 1 | (MH "Intellectual Disabilities" OR MH "Communication Disorders" OR MH "Developmental Disabilities" OR  MH "Mentally Disabled Persons" OR MH "Disabled Persons" OR AB "Disab*" OR AB "Intellectual* Disab*" OR AB "Physical* disab*" OR AB "Intellectual* Handicap*" OR AB "Intellectual* impair*" OR AB "Mental* disab*" OR AB "Physical* handicap*" OR AB "Physical* impair*" OR AB "Vision impair*" OR AB "Visual impair*" OR AB "Hearing impair*" OR AB "Deaf*" OR AB "Blind*" OR AB "Motor disab*" OR AB "Neuromotor disab*" OR AB "Mobility impair*" OR AB "Functional* impair*") |
| 2 | (MH "Domestic Violence") OR (MH "Intimate Partner Violence") OR (MH "Battered Women") OR (MH "Violence") OR (MH "Aggression") OR (MH "Spouse Abuse") OR (MH "Physical Abuse") OR (MH "Rape") OR AB "Assault" OR AB "Sexual abuse" OR AB "Sexual assault" OR AB "Psychological abuse" OR AB "Psychological violence" OR AB "Emotional abuse" OR AB "Emotional violence" OR AB "Neglect" OR AB "Economic abuse" OR AB "Financial abuse" OR AB "Verbal abuse" OR AB "Violence against women" OR AB "Abused women" OR AB "Intimate terrorism" OR AB "Marital rape" OR AB "Wife beating" OR AB "Relationship aggression" OR AB "Restraint" OR AB "Reproductive coercion" OR AB "Pregnancy coercion" OR AB "Contraceptive coercion" OR AB "Forced contraception" OR AB "Forced sterilization" OR AB "Forced sterilisation" |
| 3 | (MH "Prevalence") OR (MH "Incidence") OR AB "Prevalence" OR AB "Incidence" OR AB "Risk*" OR AB "Experienc*" OR AB "Expos*" |
| 4 | (MH "Women") OR (MH "Female+") OR AB "Wife" OR (MH "Spouses") OR AB "Wives" OR AB "Partner*" OR AB "Spouse*" |
| 1 AND 2 AND 3 AND 4 |  |
| **ASSIA, ERIC, International Bibliography of the Social Sciences (IBSS), National Criminal Justice Reference System Abstracts Database, Sociological Abstracts, Social Sciences Citation Index** – [ProQuest] | |
| 1 | AB (“disab*” OR "intellectual disability" OR "communication disorders" OR "developmental disabilities" OR  "disabled persons" OR "physical* disab*" OR "intellectual* handicap*" OR "intellectual* impair*" OR "mental* disab*" OR "physical* handicap*" OR "physical* impair*" OR "vision impair*" OR "visual impair*" OR "hearing impair*" OR "deaf*" OR "blind*" OR "motor disab*" OR "neuromotor disab*" OR "mobility impair*" OR "functional* impair*") |
| 2 | AB ("domestic violence" OR "intimate partner violence" OR "battered women" OR "violence" OR "aggression" OR "spouse abuse" OR "physical abuse" OR "rape" OR "assault" OR "sexual abuse" OR "sexual assault" OR "psychological abuse" OR "psychological violence" OR "emotional abuse" OR "emotional violence" OR "neglect" OR "economic abuse" OR "financial abuse" OR "verbal abuse" OR "violence against women" OR "abused women" OR "intimate terrorism" OR "marital rape" OR "wife beating" OR "relationship aggression" OR "restraint" OR "reproductive coercion" OR "pregnancy coercion" OR "contraceptive coercion" OR "forced contraception" OR "forced sterilization" OR "forced sterilisation") |
| 3 | AB ("prevalence" OR "incidence" OR "risk*" OR "experienc*" OR "expos*") |
| 4 | AB ("women" OR "female" OR "wife" OR "spouses" OR "wives" OR "partner*" OR "spouse*") |
| 1 AND 2 AND 3 AND 4 |  |
| **Cochrane Library** | |
| 1 | ti,ab,kw: (“Disab*” OR "Intellectual* Disab*" OR "Physical* disab*" OR "Intellectual* Handicap*" OR "Intellectual* impair*" OR "Mental* disab*" OR "Physical* handicap*" OR "Physical* impair*" OR "Vision impair*" OR "Visual impair*" OR "Hearing impair*" OR “Deaf*” OR “Blind*” OR "Motor disab*" OR "Neuromotor disab*" OR "Mobility impair*" OR "Functional* impair*") OR [MeSH descriptor: "Intellectual Disability" explode all trees] OR [MeSH descriptor: "Communication Disorders" explode all trees] OR [MeSH descriptor: "Developmental Disabilities" explode all trees] OR [MeSH descriptor: "Mentally Disabled Persons" explode all trees] OR [MeSH descriptor: "Disabled Persons" explode all trees] |
| 2 | ti,ab,kw: ("Assault" OR "Sexual abuse" OR "Sexual assault" OR Rape OR "Psychological abuse" OR "Psychological violence" OR "Emotional abuse" OR "Emotional violence" OR Neglect OR "Economic abuse" OR "Financial abuse" OR "Verbal abuse" OR "Violence against women" OR "Abused women" OR "Intimate terrorism" OR "Marital rape" OR "Wife beating" OR "Relationship aggression" OR “Restraint” OR "Reproductive coercion" OR "Pregnancy coercion" OR "Contraceptive coercion" OR "Forced contraception" OR "Forced sterilization" OR "Forced sterilisation") OR [MeSH descriptor: "Domestic Violence" explode all trees] OR [MeSH descriptor: "Intimate Partner Violence" explode all trees] OR [MeSH descriptor: "Battered Women" explode all trees] OR [MeSH descriptor: "Violence" explode all trees] OR [MeSH descriptor: "Aggression" explode all trees] OR [MeSH descriptor: "Spouse Abuse" explode all trees] OR [MeSH descriptor: "Physical Abuse" explode all trees] OR [MeSH descriptor: "Rape" explode all trees] OR [MeSH descriptor: "Restraint, Physical" explode all trees] |
| 3 | ti,ab,kw: (Prevalence OR Incidence OR Risk* OR Experienc* OR Expos*) OR [MeSH descriptor: "Prevalence" explode all trees] OR [MeSH descriptor: "Incidence" explode all trees] |
| 4 | ti,ab,kw: (Wife OR Wives OR Partner* OR Spouse*) OR [MeSH descriptor: "Women" explode all trees] OR [MeSH descriptor: "Female" explode all trees] OR [MeSH descriptor: "Spouses" explode all trees] |
| 1 AND 2 AND 3 AND 4 |  |
| **Embase** | |
| 1 | exp disabled person/ OR exp intellectual disability/ OR exp communication disorder/ OR  exp developmental disorder/ OR exp physical disability/ OR exp mentally disabled person/ OR  exp vision disorder/ OR exp hearing disorder/ OR exp motor dysfunction/ OR exp mobility limitation/ OR  (disab* OR "intellectual disability" OR "communication disorders" OR "developmental disabilities" OR  "disabled persons" OR "physical* disab*" OR "intellectual* handicap*" OR "intellectual* impair*" OR  "mental* disab*" OR "physical* handicap*" OR  "physical* impair*" OR "vision impair*" OR "visual impair*" OR "hearing impair*" OR "deaf*" OR "blind*" OR  "motor disab*" OR "neuromotor disab*" OR "mobility impair*" OR "functional* impair*").ti,ab,kw |
| 2 | exp domestic violence/ OR exp spouse abuse/ OR exp partner violence/ OR exp physical abuse/ OR  exp sexual assault/ OR exp emotional abuse/ OR exp psychological abuse/ OR exp economic abuse/ OR  exp coercion/ OR exp rape/ OR exp assault/ OR exp aggression/ OR  ("intimate partner violence" OR "battered women" OR "violence" OR "aggression" OR "spouse abuse" OR  "physical abuse" OR rape OR assault OR "sexual abuse" OR "sexual assault" OR "psychological abuse" OR  "psychological violence" OR "emotional abuse" OR "emotional violence" OR "neglect" OR "economic abuse" OR  "financial abuse" OR "verbal abuse" OR "violence against women" OR "abused women" OR "intimate terrorism" OR  "marital rape" OR "wife beating" OR "relationship aggression" OR "restraint" OR "reproductive coercion" OR  "pregnancy coercion" OR "contraceptive coercion" OR "forced contraception" OR "forced sterilization" OR  "forced sterilisation").ti,ab,kw |
| 3 | exp prevalence/ OR exp incidence/ OR exp risk/ OR exp disease incidence/ OR  (prevalence OR incidence OR risk* OR experienc* OR expos*).ti,ab,kw |
| 4 | exp female/ OR exp woman/ OR exp girl/ OR exp wife/ OR exp spouse/ OR (female OR women OR woman OR girl* OR wife OR wives OR spouse* OR partner*).ti,ab,kw |
| 1 AND 2 AND 3 AND 4 |  |
| **WHO Global Health Library (LILACS, IBCS, BDENF, African Index Medicus, Index Medicus for Eastern Mediterranean Region (IMEMR), Latin American and Caribbean Center on Health Sciences Information (PAHO) Library** | |
| 1 | ti,ab,kw: (“Disab*” OR "Intellectual* Disab*" OR "Physical* disab*" OR "Intellectual* Handicap*" OR "Intellectual* impair*" OR "Mental* disab*" OR "Physical* handicap*" OR "Physical* impair*" OR "Vision impair*" OR "Visual impair*" OR "Hearing impair*" OR “Deaf*” OR “Blind*” OR "Motor disab*" OR "Neuromotor disab*" OR "Mobility impair*" OR "Functional* impair*") OR [DeCS descriptor: "Intellectual Disability" explode all] OR [DeCS descriptor: "Communication Disorders" explode all] OR [DeCS descriptor: "Developmental Disabilities" explode all] OR [DeCS descriptor: "Mentally Disabled Persons" explode all] OR [DeCS descriptor: "Disabled Persons" explode all] |
| 2 | ti,ab,kw: ("Assault" OR "Sexual abuse" OR "Sexual assault" OR Rape OR "Psychological abuse" OR "Psychological violence" OR "Emotional abuse" OR "Emotional violence" OR Neglect OR "Economic abuse" OR "Financial abuse" OR "Verbal abuse" OR "Violence against women" OR "Abused women" OR "Intimate terrorism" OR "Marital rape" OR "Wife beating" OR "Relationship aggression" OR Restraint OR "Reproductive coercion" OR "Pregnancy coercion" OR "Contraceptive coercion" OR "Forced contraception" OR "Forced sterilization" OR "Forced sterilisation") OR [DeCS descriptor: "Domestic Violence" explode all] OR [DeCS descriptor: "Intimate Partner Violence" explode all] OR [DeCS descriptor: "Battered Women" explode all] OR [DeCS descriptor: "Violence" explode all] OR [DeCS descriptor: "Aggression" explode all] OR [DeCS descriptor: "Spouse Abuse" explode all] OR [DeCS descriptor: "Physical Abuse" explode all] OR [DeCS descriptor: "Rape" explode all] |
| 3 | ti,ab,kw: (Prevalence OR Incidence OR Risk* OR Experienc* OR Expos*) OR [DeCS descriptor: "Prevalence" explode all] OR  [DeCS descriptor: "Incidence" explode all] |
| 4 | ti,ab,kw: (Wife OR Wives OR Partner* OR Spouse*) OR [DeCS descriptor: "Women" explode all] OR [DeCS descriptor: "Female" explode all] OR [DeCS descriptor: "Spouses" explode all] |
| 1 AND 2 AND 3 AND 4 |  |
| **Western Pacific (WPRO)** | |
| 1 | ti,ab,kw: (“Disab*” OR "Intellectual* Disab*" OR "Physical* disab*" OR "Intellectual* Handicap*" OR "Intellectual* impair*" OR "Mental* disab*" OR "Physical* handicap*" OR "Physical* impair*" OR "Vision impair*" OR "Visual impair*" OR "Hearing impair*" OR “Deaf*” OR “Blind*” OR "Motor disab*" OR "Neuromotor disab*" OR "Mobility impair*" OR "Functional* impair*") |
| 2 | ti,ab,kw: ("Assault" OR "Sexual abuse" OR "Sexual assault" OR Rape OR "Psychological abuse" OR "Psychological violence" OR "Emotional abuse" OR "Emotional violence" OR Neglect OR "Economic abuse" OR "Financial abuse" OR "Verbal abuse" OR "Violence against women" OR "Abused women" OR "Intimate terrorism" OR "Marital rape" OR "Wife beating" OR "Relationship aggression" OR "Restraint" OR "Reproductive coercion" OR "Pregnancy coercion" OR "Contraceptive coercion" OR "Forced contraception" OR "Forced sterilization" OR "Forced sterilisation") |
| 3 | ti,ab,kw: (Prevalence OR Incidence OR Risk* OR Experienc* OR Expos*) |
| 4 | ti,ab,kw: (women OR woman OR female* OR wife OR wives OR partner* OR spouse*) |
| 1 AND 2 AND 3 AND 4 |  |
