# Supplementary material for: Global prevalence of violence against women with disabilities: protocol for a systematic review and meta-analysis
Source: BMJ Open. 2025 Nov 4;15(11):e102746. doi: 10.1136/bmjopen-2025-102746 (PMC12588038; doi:10.1136/bmjopen-2025-102746)
Supplement: Supplementary file 2 [file bmjopen-2025-102746.reviewer_comments.pdf]

PEER REVIEW HISTORY

BMJ Open publishes all reviews undertaken for accepted manuscripts. Reviewers are asked to complete a checklist review form and are provided with free text boxes to elaborate on their assessment. These free text comments are reproduced below.

ARTICLE DETAILS

Title (Provisional)

Global Prevalence of Violence Against Women with Disabilities: Protocol for a Systematic Review and Meta-Analysis

Authors

Campo Tena, Laura; Sardinha, LynnMarie; Meyer, Sarah R.; Garcia-Moreno, Claudia

---

VERSION 1 - REVIEW

Reviewer 1

Name Eldred, Emily

Affiliation London School of Hygiene and Tropical Medicine

Date 19-Jun-2025

COI I am submitting a joint bid for a conference workshop with one of the authors. We have not previously worked together.

Otherwise, I report no competing interests.

Thank you for the opportunity to review this manuscript. The protocol is thorough, clearly setting out the review methodology. The systematic review topic will fill an important gap in the field of VAW research, adding sex-disaggregated prevalence data on violence against women with disabilities that has not previously been synthesised. I outline minor suggestions below:

• Search terms:

o The authors might want to also include ‘disab*’ to account for references to ‘disabled’ and ‘disability’. ‘Autism’ is often referred to separately in the literature so the authors might consider adding that as a separate term depending on whether this fits with their definition of disability

o for search terms on violence the authors might also want to consider items on reproductive coercion which may be particularly relevant to women with disabilities, and other terms may need to be added contingent on the point outlined below

• The research question ‘1) What are the global prevalence estimates for VAW with disabilities?’ and the extraction data ‘Type of violence reported (physical, psychological, sexual, any violence, 203 other)… Type of perpetrator (e.g., intimate or dating partner, spouse, ex-intimate 205 or ex-dating partner, ex-spouse, relative, formal caregiver, any)’ suggests that the review will be looking at a broad range of types of violence across different perpetrators. However, in the introduction, search terms and inclusion criteria, this is restricted to ‘violence by intimate partners and other family members or caregivers, as well as sexual violence by any perpetrator.’ It would be useful to standardise this throughout the manuscript. Also noting that the latter is similar to previously published literature.

**Reviewer**	**2**
**Name**	**Alkan, Ömer**
**Affiliation**	**Ataturk University, Department of Econometrics**
**Date**	**04-Aug-2025**
**COI**	**None**

I have carefully reviewed this manuscript and below is my decision.

- In Conclusion section, I recommend highlighting the most relevant contributions of the study and proposing concrete recommendations for future research or training programmes. A section on future research directions could be included.

-I would suggest adding to the literature and referencing it within the introduction and discussion as well. There are studies that have examined violence.

1) https://doi.org/10.1007/s10389-023-01999-x

2) https://doi.org/10.1177/21582440241251989

3) https://doi.org/10.1186/s12905-021-01333-1

4) https://doi.org/10.3389/fpsyg.2023.1174143

5) https://doi.org/10.1186/s12889-025-22009-7

6) https://doi.org/10.1371/journal.pone.0248630

7) https://doi.org/10.1371/journal.pone.0275950

- The topic is quite interesting, however, the explanation on the originality of the study is insufficient. This paper, needs to highlight clearly the originality of the study.

- How does the paper contribute to the extant literature on the subject?

It is ready for publication once the necessary corrections are made.

VERSION 1 - AUTHOR RESPONSE

Reviewers' Comments	Response
**Reviewer 1**	
Search terms: The authors might want to also include 'disab*' to account for references to 'disabled' and 'disability'. 'Autism' is often referred to separately in the literature so the authors might consider adding that as a separate term depending on whether this fits with their definition of disability<br>o for search terms on violence the authors might also want to consider items on reproductive coercion which may be particularly relevant to women with disabilities, and other terms may need to be added contingent on the point outlined below	Thank you for your suggestions regarding the search terms. We conducted a preliminary search with an adjusted strategy based on your feedback. Given the substantial number of results obtained in the updated search, we have incorporated the recommended terms that include "disab*". We also added additional terms on reproductive coercion, considering their relevance to the study of VAW among people with disabilities.<br>Regarding the inclusion of autism-related terms, we conducted a preliminary screening in PubMed to inform our decision. Of the references retrieved with the adjusted strategy, 503 included the term "Autis*". These were screened by title and abstract. From this set, only four were taken forward for full-text review: two were excluded, one could not be accessed, and one remains uncertain and requires further discussion. Given this very low yield and limited relevance to our operational definition of disability, we have decided not to include autism-specific terms in the main search.<br><br>*Pages 11-12*

The research question ‘1) What are the global prevalence estimates for VAW with disabilities?’ and the extraction data ‘Type of violence reported (physical, psychological, sexual, any violence, 203 other)… Type of perpetrator (e.g., intimate or dating partner, spouse, ex-intimate or ex-dating partner, ex-spouse, relative, formal caregiver, any)’ suggests that the review will be looking at a broad range of types of violence across different perpetrators. However, in the introduction, search terms and inclusion criteria, this is restricted to ‘violence by intimate partners and other family members or caregivers, as well as sexual violence by any perpetrator.’ It would be useful to standardise this throughout the manuscript. Also noting that the latter is similar to previously published literature.	Thank you for highlighting this inconsistency. We have now standardized the description of the study scope across the introduction, research questions, data extraction fields, and inclusion criteria. Specifically, we clarified that the review will consider physical, psychological, sexual, and other forms of violence (including neglect and sexual/reproductive coercion), while focusing on the following broad categories: intimate partner violence, domestic violence (primarily family members), and sexual violence by any perpetrator (covering various potential perpetrators, and including caregivers).<br><br>In addition, one of the reasons for extracting detailed data, for example, on type of perpetrator is to screen studies for eligibility and, where feasible, to allow for potential future in-depth analyses that may go beyond the primary scope of this review. We have now specified this.<br><br>*Page 8, Lines 113-120*<br>*Page 9, Lines 128-133*<br>*Page 15, Lines 216-217*<br>*Page 18, Lines 295 to Page 19, Line 308*
**Reviewer 2**	
In Conclusion section, I recommend highlighting the most relevant contributions of the study and proposing concrete recommendations for future research or training programmes. A section on future research directions could be included.	We thank the reviewer for this valuable suggestion. We have revised the conclusion to explicitly highlight the anticipated contributions of our study and to outline concrete recommendations for future research and training programmes. We have added a section that emphasizes the importance of including sex-disaggregated data, broadening the scope of studied abuse beyond intimate partner violence, and strengthening methodological and training approaches to

Reviewers’ Comments	Response
	ensure more comprehensive and inclusive evidence in this field.<br><br>*Page 20, Lines 328-338*

I would suggest adding to the literature and referencing it within the introduction and discussion as well. There are studies that have examined violence.<br><br>1) https://doi.org/10.1007/s10389-023-01999-x<br>2) https://doi.org/10.1177/21582440241251989<br>3) https://doi.org/10.1186/s12905-021-01333-1<br>4) https://doi.org/10.3389/fpsyg.2023.1174143<br>5) https://doi.org/10.1186/s12889-025-22009-7<br>6) https://doi.org/10.1371/journal.pone.0248630<br>7) https://doi.org/10.1371/journal.pone.0275950	We thank the reviewer for suggesting additional literature. We carefully reviewed the recommended publications. While these studies provide valuable insights into violence against women, most of them focus on a specific country context (i.e., Turkey) and do not directly align with the global scope or disability-specific focus of our review. For this reason, we determined that citing them extensively would not be fully relevant to our research objectives. Nevertheless, we have incorporated a relevant point from one of these papers to highlight how poor or very poor health status can increase women's vulnerability to violence, as this complements our discussion. We believe this integration maintains consistency with the aims and scope of our study.<br><br>*Page 6, Lines 60-62*
The topic is quite interesting, however, the explanation on the originality of the study is insufficient. This paper, needs to highlight clearly the originality of the study.	Thank you for this comment. We now emphasize that, unlike previous reviews that were limited to adults with no sex-disaggregated data, or focused solely on intimate partner violence, our study will be the first systematic review and meta-analysis to comprehensively assess the global prevalence and risk of violence against women with disabilities. We underline its originality in explicitly incorporating sex-disaggregated data, examining multiple forms of violence by different perpetrators, and applying rigorous quantitative synthesis methods.<br><br>*Page 8, Lines 113-120*
How does the paper contribute to the extant literature on the subject?	Thank you for this insightful comment. In response, we discuss more in detail how our study advances the existing literature. We now emphasize that, unlike earlier reviews that were limited in scope, this systematic review and meta-analysis will

Reviewers’ Comments	Response
	be the first to provide a comprehensive and quantitative synthesis of global prevalence and risk of violence against women with disabilities. By integrating multiple forms of violence and systematically incorporating sex-disaggregated data, our review contributes novel evidence that can inform both future research and applied policy and practice.<br><br>*Page 8, Lines 113-120*<br>*Page 18, Lines 295 to Page 19, Line 308*<br>*Page 20, Lines 328-338*

VERSION 2 - REVIEW

Reviewer 1

Name Eldred, Emily

Affiliation London School of Hygiene and Tropical Medicine

Date 29-Sep-2025

COI

Thank you for
